# Assessment of the real-world impact of the Thai smoking cessation programme on clinical outcomes: protocol for a multicentre prospective observational study

**DOI:** 10.1017/S1463423622000548

**Published:** 2022-11-10

**Authors:** Chayutthaphong Chaisai, Nathorn Chaiyakunapruk, Kednapa Thavorn, Somkiat Wattanasirichaigoon, Suthat Rungruanghiranya, Araya Thongphiew, Shaun Wen Huey Lee

**Affiliations:** 1 School of Pharmacy, Monash University Malaysia, Selangor, Malaysia; 2 Department of Pharmaceutical Care, Faculty of Pharmacy, Chiang Mai University, Chiang Mai, Thailand; 3 Department of Pharmacotherapy, College of Pharmacy, The University of Utah, Salt Lake City, UT, USA; 4 School of Epidemiology and Pubic Health, University of Ottawa, Ottawa, ON, Canada; 5 Ottawa Hospital Research Institute, Ottawa, ON, Canada; 6 Thai Physician Alliance Against Tobacco, Bangkok, Thailand; 7 Faculty of Medicine, Srinakharinwirot University Ongkharak, Nakornnayok, Thailand; 8 Paolo Phaholyothin Hospital (BDMS), Bangkok, Thailand; 9 School of Pharmacy, Taylor’s University, Selangor, Malaysia

**Keywords:** smoking cessation, smoking abstinence, prevalence, protocol, multidisciplinary team

## Abstract

**Background:**

Tobacco smoking is the most common preventable cause of morbidity and mortality in the world. In an effort to counteract the harmful consequences of smoking, various tobacco control measures have been implemented, including the use of smoking cessation programmes to reduce the number of new smokers as well as helping current smokers to quit smoking. In Thailand, the SMART Quit Clinic Program (FAH-SAI Clinics) was launched in 2010 to provide smoking cessation services by a multidisciplinary team. There are currently 552 FAH-SAI Clinics established across all 77 provinces of Thailand.

**Aim:**

This protocol describes a study aiming to evaluate the SMART Quit Clinic Program (FAH-SAI Clinics) in terms of programme performance and clinical outcomes. We hope that the results of the study could be used to improve the current service model and the programme’s success.

**Method:**

A multicentre prospective observational study will be conducted. The study will focus on 24 FAH-SAI Clinics across 21 provinces of Thailand. The primary outcomes are seven-day point prevalence abstinence rate and continuous abstinence rate at three and six months. The outcomes will be measured using a self-reported questionnaire and biochemical validated by exhaled carbon monoxide.

**Discussion:**

This study will be the first real-world study that reports the effectiveness of the well-established smoking cessation programme in Thailand. Findings from this study can help improve the quality of smoking cessation services provided by multidisciplinary teams and other smoking cessation services, especially those implemented in low- and middle-income countries.

## Introduction

Tobacco smoking is the most common preventable cause of morbidity and mortality in the world (World Health Organization 2017; 2021). Globally, tobacco-related mortality accounts for 8.2 million deaths yearly, with 7 million deaths among people who use tobacco and 1.2 million deaths due to exposure to second-hand smoke. Tobacco smoking is a risk factor for many non-communicable diseases, including cardiovascular disease, chronic obstructive pulmonary disease, cerebrovascular disease (e.g., stroke) and cancer, especially lung cancer (National Center for Chronic Disease Prevention and Health Promotion Office on Smoking Health, 2014; Gowing *et al.*, 2015; Mons *et al.*, 2015; West, 2017; World Health Organization, 2017; 2021). In addition to the health impact, smoking is also associated with productivity loss, increased healthcare spending and decreased overall quality of life (Heikkinen *et al.*, 2008; Vogl *et al.*, 2012; Coste *et al.*, 2014).

Given the consequences of tobacco smoking, reducing the number of new smokers as well as helping current smokers to quit tobacco is now a pressing global agenda (National Center for Chronic Disease Prevention and Health Promotion Office on Smoking Health, 2014; World Health Organization, 2017; 2021). Epidemiological modelling studies (Gartner *et al.*, 2009) have showed that smoking cessation can significantly reduce smoking prevalence. To date, there are several interventions to promote smoking cessation, including pharmacotherapy, behavioural support, health education promotion, mobile health apps and traditional and complementary medicine (Cahill *et al.*, 2013; Lancaster and Stead, 2017). Previous studies have shown that interventions (Aung *et al.*, 2019; Odorico *et al.*, 2019; World Health Organization, 2021), which incorporate self-help strategies alone without face-to-face counselling, were less effective compared with face-to-face counselling, while programmes with brief advice intervention, defined as providing a short individual advice and information how to quit smoking, showed a 4–11% higher continuous abstinence at 12 months of follow-up compared with usual care. Smoking cessation programmes that included multiple session behavioural change strategies such as a series of meeting with smokers were more effective than usual care and brief advice (World Health Organization, 2017; Aung *et al.*, 2019; Odorico *et al.*, 2019; World Health Organization, 2021).

Currently, only 26 countries, which account for one-third of the world population, have national comprehensive cessation services with full or partial cost coverage (World Health Organization, 2017). While various systematic reviews have provided evidence supporting interventions that increase smoking cessation, not all of these interventions have been adopted in many countries. Reasons include cost of services, smoking culture, client resistance and lack of trained healthcare professionals (Zapka *et al.*, 2004; Li *et al.*, 2014; Pagano *et al.*, 2016). In many low-middle income countries such as Thailand (Vathesatogkit and Charoenca, 2011), scarce resource, infrastructures and access to resources are additional barriers to implementation of such programmes.

In the past two decades, Thailand launched several smoking cessation services, for example, smoking cessation services in community pharmacies are encouraged by the Thai Pharmacy Network for Tobacco Control or Thai Health Professional Alliances Against Tobacco as well as National Alliances for Tobacco Free Thailand. Since 2010, Thailand has also launched the smoking cessation service programme under the SMART Quit Clinic Program (FAH-SAI Clinics) which provides smoking cessation services by a multidisciplinary team. A previous report by the Thailand National Alliance for Tobacco suggested that the self-reported point prevalence of smoking quit rate at three and six months was 33.9% and 38.2%, respectively (The National Alliance for Tobacco-Free Thailand, 2011). However, it remains unclear if these rates are representative of the actual success of the clinics as their real-world effectiveness has never been formally evaluated. This protocol aims to describe the evaluation process of the impact of the FAH-SAI Clinics in terms of programme performance and clinical outcomes.

The objectives of the study are

- To evaluate the impact of the FAH-SAI Clinics in terms of seven-day point prevalence abstinence rate (PAR) and smoking continuous abstinence rate (CAR) at three and six months.
- To assess factors associated with seven-day PAR, and CAR at three and six months.

## Methods and analysis

### Study design

We will conduct a multicentre prospective observational study. As the FAH-SAI Clinics have been implemented since 2010, it is considered a standard care which is provided to all smokers in Thailand. As such, it was not possible to have a control group.

### Study setting

This study will focus on 24 FAH-SAI Clinics across 13 health regions of Thailand. These clinics will be chosen based upon a stratified random sampling to reduce selection bias and ensure that the study samples are representative of the geographical regions, based upon the following criteria:

- Previous performance of each setting defined by using a recruitment rate and the number of visits in the previous year;
- Location of FAH-SAI Clinics by using Thailand 13 health regional strata.

**Figure 1.**
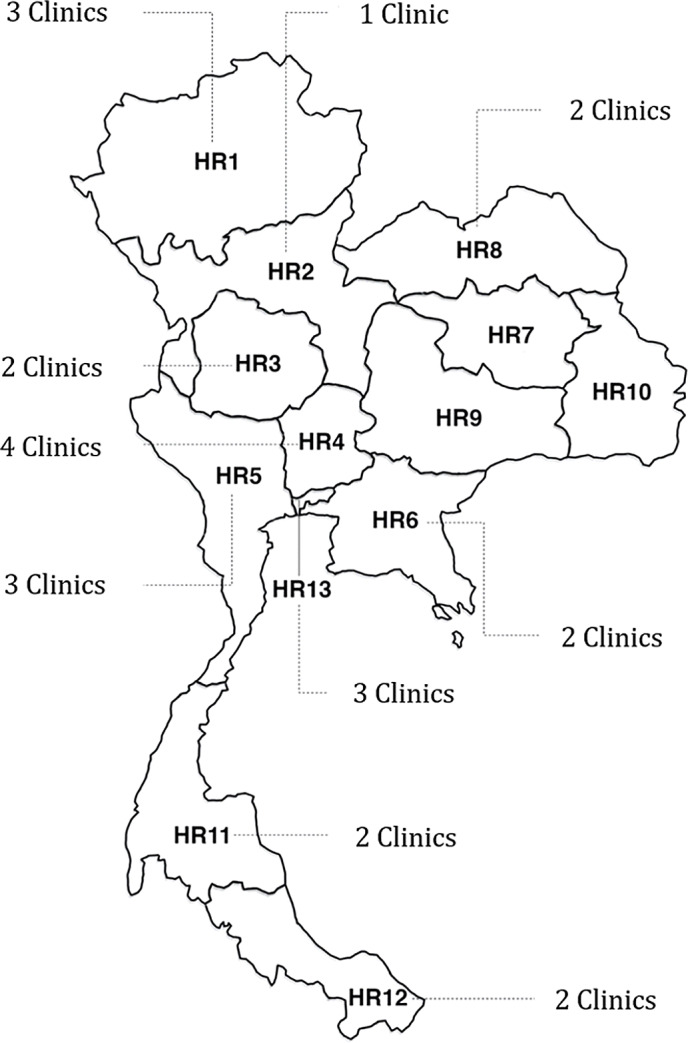
Map of Thailand health regions and the number of FAH-SAI Clinics in the study (HR: Health region or Health district).

In this study, the samples will be drawn from 2 university hospitals, 10 tertiary hospitals, 11 secondary hospitals and 1 private hospital (Figure 1).

### Study population

#### Inclusion criteria

Participants aged 13 years or older who are in the contemplation or action stage and who have not attended or participated in any smoking cessation programme in the FAH-SAI Clinics are eligible to participate in this study.

#### Exclusion criteria

Participants will be excluded from the study if they are diagnosed with any cancers or intellectual disability that may impair the completion of the testing measure at the time of screening, have missing information on the number of cigarettes per day, have no clear history of the severity of nicotine dependent, cannot access exhaled carbon monoxide (CO) test during the follow-up periods or refuse to participate in the study.

### Procedure

#### Recruitment

All participants presenting at the FAH-SAI Clinics will be screened for eligibility by a healthcare professional at each clinic. Eligible participant and their caregiver will be invited to participate in the study. All participants will be required to sign a written consent prior to enrolment. If the participant is aged 18 years and below, consent will be obtained from their legal parents/caregiver. We will also record the reason for exclusion and refusal.

#### The FAH-SAI Clinics

The FAH-SAI Clinics Program was developed by the National Alliance for Tobacco-Free Thailand in 2010 to provide a comprehensive smoking cessation service to all Thai citizens. The programme is fully funded by the Thai Health Promotion Foundation, and all eligible citizens receive the service for free. Currently, there are 552 healthcare facilities that have joined the network covering 77 provinces across Thailand. The FAH-SAI Clinics is mainly operated by trained nurses who will consult with the attending physician if needed.

#### Standard interventions

At the FAH-SAI Clinics, the interventions and activities are standardised based upon a protocol developed by the Ministry of Health and National Alliance for Tobacco-Free Thailand. It follows the well-established 5As model in smoking cessation (Ask, Advise, Assess, Assist and Arrange). Activities include:

- Identify, diagnose and document tobacco use status
- Assess for the severity of nicotine dependence status
- Advise the patient to quit smoking
- Assess for patient’s that willing to quit smoking
- Assist the patient to quit smoking using counselling techniques together with pharmacological methods such as nicotine replacement therapy, herbs and traditional therapy
- Schedule follow-up with patient either in-person, through telephone contact or social network.

Interventions and activities vary slightly across settings depending on local context and availability of human resources. For instance, home visits may be arranged in some settings while in others only group counselling is conducted. Each session will typically last between 15 and 30 minutes, with follow-up at months 1, 3 and 6.

#### Initial assessments (VISIT 0)

In the initial assessment (VISIT 0), participants will be interviewed by a healthcare professional to collect socio-demographic data including age, gender, marital status, occupation, level of education, income, reimbursement scheme and comorbidities. We will also identify and document tobacco use status, nicotine dependence status based upon the number of cigarette smoked and Fagerstrom test for nicotine dependence, measure exhaled CO or other tests if applicable. All participants will then be provided with the standard intervention described above.

#### Follow-up assessments

**Figure 2.**
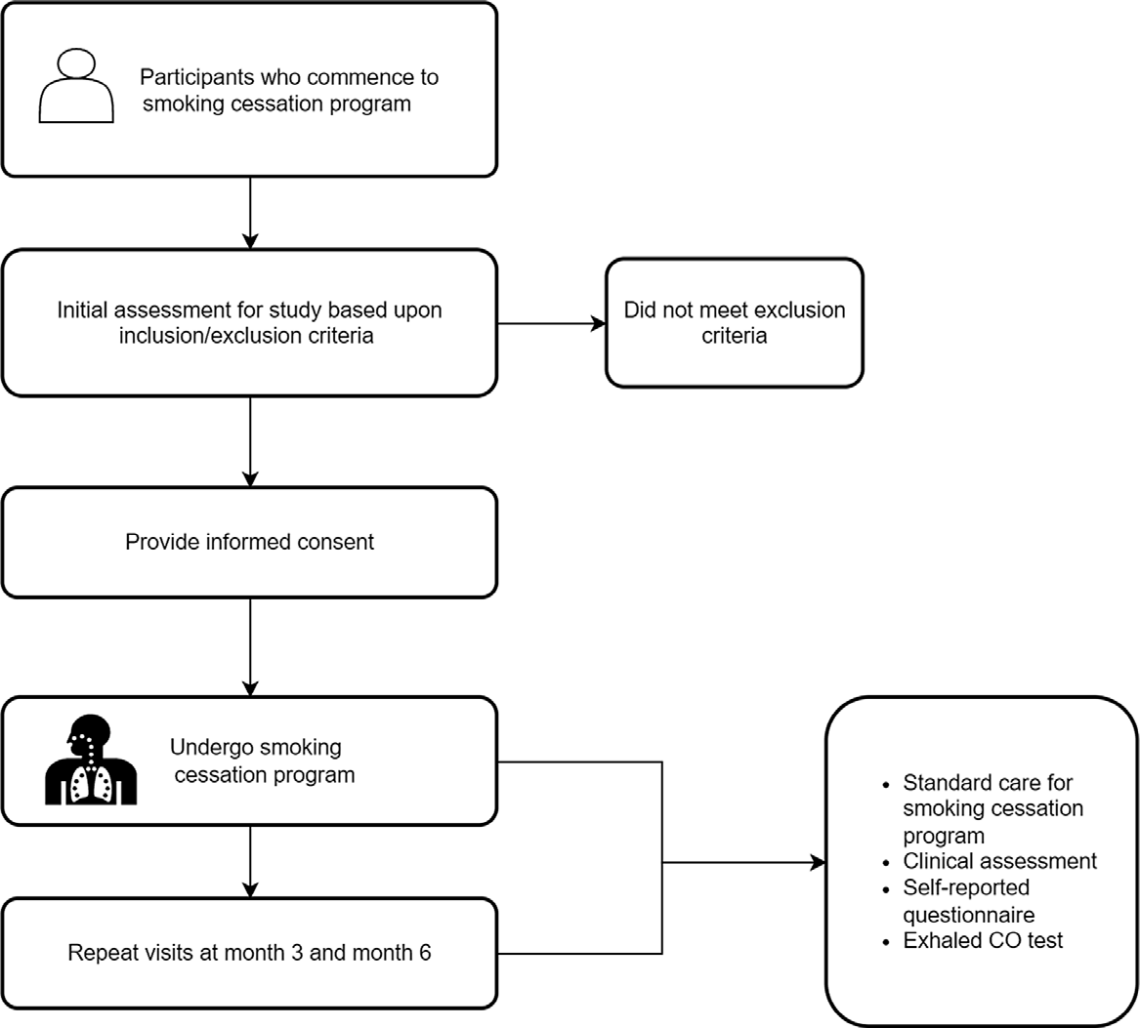
Flow chart of the study assessment.

Following the initial assessment, all participants will be asked to visit the FAH-SAI Clinics for follow-up. These appointments include research and clinical assessment performed at three and six months following the baseline visit. At each follow-up visit, participant will receive standard intervention and will be assessed on the progression of smoking cessation. Tests performed include Fagerstrom test for nicotine dependence, exhaled CO test and a self-reported questionnaire to identify seven-day PAR, number of cigarettes smoked in seven days, and CAR at three and six months. The healthcare professional will evaluate and monitor any adverse events associated with nicotine replacement therapy (Table 1 and Figure 2).

**Table 1.** Summary of data collection and timeline

Timeline	VISIT 0 Baseline	VISIT 1 3 months	VISIT 2 6 months
Demographic	✓		
Self-reported questionnaire			
• Seven-day point prevalence abstinence	✓	✓	✓
• Continuous abstinence rate	✓	✓	✓
Exhaled carbon monoxide test			
• Seven-day point prevalence abstinence	✓	✓	✓
• Continuous abstinence rate	✓	✓	✓
Fagerstrom test for nicotine dependence	✓	✓	✓
Smoking cessation-related activities in FAH-SAI Clinics	✓	✓	✓

### Outcome measures

The primary outcome of this study is smoking CAR at three and six months. Secondary outcomes are seven-day PAR at three and six months. Factors that are associated with smoking CAR will be investigated.

To evaluate the outcomes, we will use two measurement methods: (1) a self-reported questionnaire and (2) exhaled CO levels, which is currently the gold standard for biochemical validation method. Exhaled CO levels will be evaluated using the CO-oximeter, which will be calibrated and validated before each evaluation. At the given point in time (three and six months), participants will be asked whether they have used any form of tobacco in the past. CAR will be measured using the question: ‘When did you quit smoking?’ and ‘Have you used any tobacco (any type) after the quit date?’. The quit date will be recorded and those who reply that they have not used any form of tobacco after the quit date and have fixing CO concentrations in exhaled air to be under 10 ppm are considered to have quit (depending on the duration).

Seven-day PAR will be evaluated using a global evaluation question: ‘Have you used any tobacco (any type) in the last 7 days?’. Those who reply that they have not used any form of tobacco in the past 7 days and have fixing CO concentrations in exhaled air to be under 10 ppm are considered to have quit at the follow-up visit (three and six months).

### Data analysis plan

Descriptive statistics (mean, SD, median, IQR, percentage) will be used to describe the baseline characteristics of participants in this study. Baseline characteristics will be assessed whether they are associated with the three- and six-month smoking CAR of patients by using logistic regression. Age, gender, socioeconomic status and comorbidities will be controlled in the analysis. All data will be analysed by using STATA version 14.0 (College Station, TX).

### Sample size and power calculation

Based upon a previous report by the FAH-SAI Clinics (The National Alliance for Tobacco-Free Thailand, 2011), the smoking cessation success rate at six months was 38.2%. Assuming an alpha level of 0.05, with 80% power, and accounting for a 10% dropout rate, we would require a minimum of 1,540 participants.

### Ethics and dissemination

The study was approved by the Ethical Review Committee for Research in Human Subjects, Ministry of Public Health, Thailand (Protocol number 24/2562 and document number 51/2019). The study will be conducted in accordance with the Declaration of Helsinki, participation is voluntary and written informed consent will be obtained. The study poses little to no risk on participants and their caregiver. Participation in this study does not interfere with the typical care of smoking patients. The result of the study will be published in peer-reviewed journals and presented at research, clinical and public conferences.

## Discussion

Smoking cessation programmes have been found to successfully reduce the number of new smokers and mitigate the long-term health problems due to smoking. However, the real-world evaluation of the programme should be conducted to ensure that the programme offers high quality and value of care. To address this gap, our study will evaluate the impact of smoking cessation programme in Thailand, namely FAI-SAI Clinic, in terms of its programme performance and clinical outcomes by using an observational study approach to reflex clinical outcome and programme performance in real-world settings. Additionally, we will use the exhaled CO levels as a biochemical confirmation to ensure that the study’s findings accurately reflect the true effectiveness of the smoking cessation programme. The use of a real-world observational study design is advantageous in this instance, as we will be able to estimate the true effects of the public health initiative as a whole.

Our study will also add to the body of knowledge regarding the real-world evidence on smoking cessation programmes. Previous studies have reported varying levels of compliance and success rates of smoking cessation programmes, ranging from as low as 21% to as high as 60% due to a myriad of reasons including health system priorities, healthcare providers attitude and patient attitude. For example, in Turkey studies (Saylan *et al.*, 2021) have reported smoking cessation interventions with quit rates between 22% and 51%. In Malaysia (Zamzuri *et al.*, 2021), they developed a structured programme using a private public partnership (mQuit) with success rates of approximately 30%. Through this study, we believe that our results will provide policy makers especially in low-middle income countries on the effectiveness of such programmes and how these can be adapted to suit their context. Our results will provide a high-quality evidence, as previous studies conducted to date only used the self-reported point PAR or the CO point PAR, but not both (Kotz *et al.*, 2014; Poulsen *et al.*, 2015; Lertsinudom *et al.*, 2021).

However, as this study is observational in nature, there are several limitations associated with this design including temporal ambiguity and selection bias. Indeed, as the coverage of the FAH-SAI Clinics and its coverage is unknown, we are unable to ascertain if the population recruited in this study is truly representative of the Thailand population. To account for this, we will use a stratified random sampling technique to reduce selection biases. Second, activities at each of the FAH-SAI Clinics vary, depending on their contexts and the availability of resources. This variation may affect the performance of the smoking cessation programme. To minimise this bias, we used a stratified random sampling method taking into account previous setting performance and health regional strata in an attempt to improve the generalisability of the results as well as to represent all the health regions across the country. Importantly, this study does not include a control group, as the FAH-SAI Clinics is considered to provide standard care to all smokers. As such, it is not possible to have a control group who will not receive smoking cessation service as comparison.

## Conclusion

This paper describes the study design for the evaluation of a national smoking cessation programme that is implemented in Thailand. The results of this study aim to provide insight into the performance and effectiveness of the programme, which can be used to improve the current service model and guide future public policy making in Thailand. The effectiveness of this programme will be reported in subsequent publications.
